# Sentinel lymph node identification using contrast-enhanced ultrasound in breast cancer: review of the literature

**DOI:** 10.1007/s10396-023-01313-y

**Published:** 2023-07-10

**Authors:** Kiyoka Omoto, Kazushige Futsuhara, Tamami Watanabe

**Affiliations:** 1 Department of Laboratory Medicine, Jichi Medical University, Saitama Medical Center, 1-847 Amanuma-cho, Omiya-ku, Saitama, 330-8503 Japan; 2 Department of Surgery, Jichi Medical University, Saitama Medical Center, 1-847 Amanuma-cho, Omiya-ku, Saitama, 330-8503 Japan

**Keywords:** Contrast-enhanced ultrasound (CEUS), Ultrasound contrast agent (UCA), Breast cancer, Sentinel lymph node (SLN), Sonazoid

## Abstract

Before breast cancer surgery, sentinel lymph node (SLN) identification and biopsy using blue dye, radioisotope (RI) with a gamma probe, or a combination of the two are mainly performed. The dye-guided method requires skilled technique to make an incision in the skin and identify SLNs without damaging the lymphatic vessels. In addition, dye-induced anaphylactic shock has been reported. To use the γ-probe-guided method, the facility must be able to handle RI. However, to overcome the drawbacks of these methods, Omoto et al. developed a new identification modality using contrast-enhanced ultrasound with an ultrasound contrast agent (UCA) in 2002. Since then, many basic experiments and clinical studies using various UCA have been reported. In particular, a number of studies in SLN detection using Sonazoid have been reported and are herein reviewed.

## Introduction

Contrast-enhanced ultrasound (CEUS) is a relatively simple and safe examination compared with contrast-enhanced computed tomography and magnetic resonance imaging, and it is performed in various aspects of breast cancer diagnosis and therapy. In addition to discrimination of benign from malignant tumors, it has also been used for the evaluation of tumor spread and response to therapy. Identification and biopsy of sentinel lymph nodes (SLNs) are routinely performed as a preoperative examination for breast cancer. In Japan, SLN detection methods using dyes or radioisotopes in breast cancer are covered by insurance, but the use of ultrasound contrast agents is not. Many studies have been performed using CEUS as one of the modalities in SLN detection. Here we review the literature including our own studies.

## Basic research on SLN identification using CEUS

There have been many basic research studies of SLN identification with radiological methods (e.g., scintigraphy), but a novel method using CEUS with an ultrasound contrast agent (UCA) was developed by Omoto et al. in 2002 [1]. They reported that when 25% albumin was injected into a pig's neck, contrast-enhanced regional lymph nodes (LNs) were visualized and SLNs could be identified. In 2004, Goldberg et al. [2] reported SLN detection using CEUS with Sonazoid (GE Healthcare, Tokyo, Japan) in swine models of melanoma. The enhanced LNs and lymphatic channels were also visualized by using CEUS with Sonazoid, and the accuracy of SLN detection was 90%.

In 2006, Omoto et al. [3] reported a new method consisting of a combination of hydroxyethylated starch as the UCA and 5% patent blue violet in swine models. The regional LNs were easily found on CEUS, and blue-stained LNs could be detected after the skin was cut at the site where ultrasound had shown they would be.

In 2006, Lurie et al. [4] reported a CEUS detection method for SLN with a microbubble contrast medium (Definity; Lantheus Medical Imaging, Massachusetts, USA). After it was injected into peritumoral tissue in 10 dogs with spontaneous head or neck tumors, regional LNs were imaged up to 20 min after contrast administration using power Doppler US.

In 2009, Wang et al. [5] reported gray-scale CEUS was performed in 12 rabbits with breast VX2 tumors after subcutaneous administration of SonoVue (Bracco, Milan, Italy). The sensitivity of CEUS for detecting SLNs was 89.5% (17/19). Among the 17 SLNs detected on CEUS, tumor metastases were identified histopathologically in four SLNs, whereas proliferation of lymphatic tissue was identified in the other 13 SLNs.

In 2015, Kogashiwa et al. [6] reported a tracer mixture of Sonazoid and indocianine green (ICG) for SLN detection. After it was injected into the tongue, larynx, and oropharynx and hypopharynx, the enhanced LNs could be transcutaneously identified in eight rabbits and four swines on CEUS. On the other hand, the SLNs could also be detected with ICG fluorescence using the Hyper Eye Medical System (HEMS).

Animal studies of SLN detection using CEUS with a UCA are summarized in Table 1.

**Table 1:** SLN detection using CEUS in the animal study

Authors	Year	Ultrasound contrast agent	Subjects	Number of subjects	Detection rate
Omoto K, et al. [1]	2002	5% Albumin, 25% Albumin	Pig	5,4	0% (0/5),100% (4/4)
Goldberg BB, et al. [2]	2004	Sonazoid	Swine	6	90% (28/31)
Omoto K, et al. [3]	2006	hydroxyethylate + patent blue violet	Swine	4	100% (4/4)
Lurie DM, et al. [4]	2006	Definity	Dog	10	80% (8/10)
Wang Y, et al. [5]	2009	SonoVue	Rabbit	12	89.5% (17/19)
Kogashiwa Y, et al. [6]	2015	Sonazoid + Indocianine Green	Rabbit, swine	8 rabbits, 4 swines	100% (8/8), 100% (5/5)

## Clinical study of SLN identification in breast cancer and other cancers

### Breast cancer

In 2006, Omoto et al. [7] first reported that SLNs could be identified with CEUS in breast cancer patients. CEUS using 25% albumin as a UCA was performed in 23 breast cancer patients, and enhanced LNs were identified in all.

**Fig. 1:**
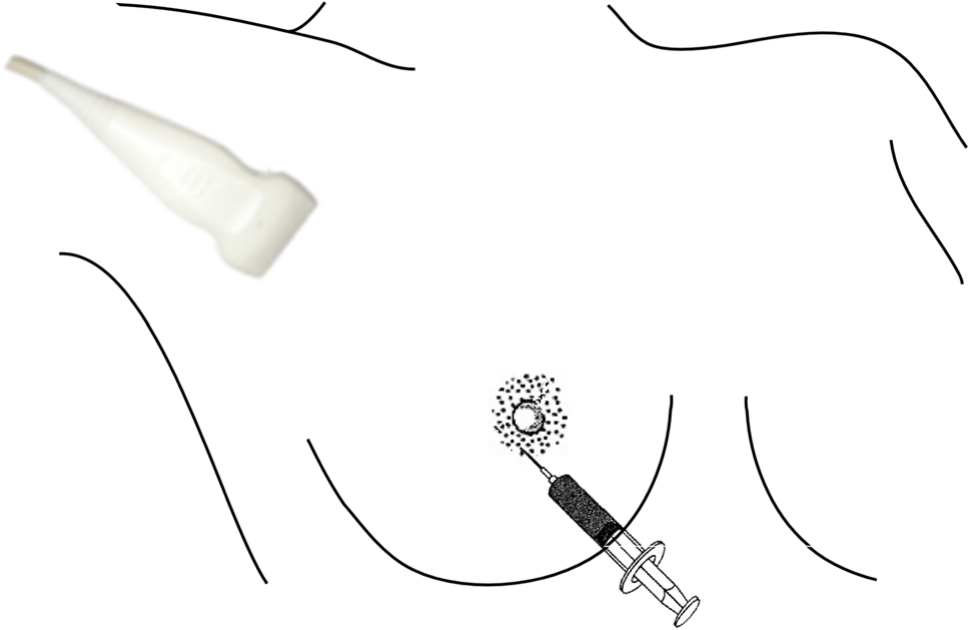
Schema of Sonazoid injection and axillary observation by CEUS in our study. To avoid high pressure, 1 ml of Sonazoid is slowly injected into the areolar margin intradermally. After gentle massage the injected area, immediately the axillary area is observed with CEUS using linear transducer

**Fig. 2:**
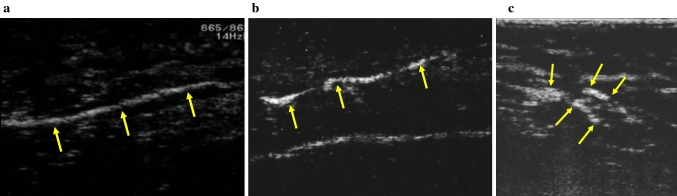
Various patterns of lymphatic flow, (**a**) An elongated lymphatic vessel (arrows) was visualized linearly within a few minutes after Sonazoid injection. In real time, lymphatic flow could be observed like a stream. (**b**) The tortuous lymph vessel (arrows) was contrast-enhanced and observed. (**c**) Branching lymphatic vessels (arrows) were observed

**Fig. 3:**
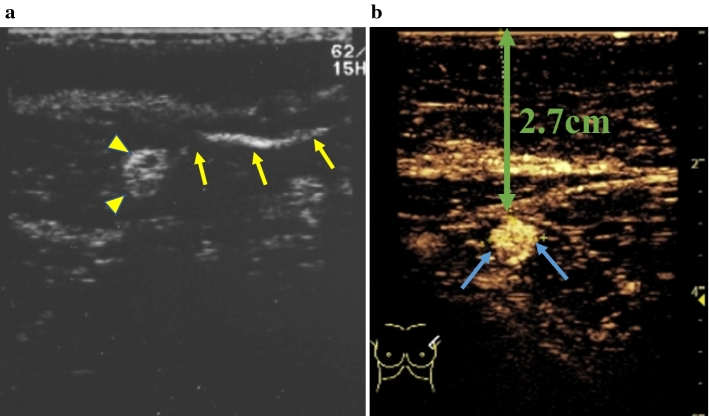
Contrast-enhanced SLN, (**a**) Following the end of the lymphatic vessel (arrows), an oval-shaped contrast-enhanced lymph node (arrowheads) was easily identified. (**b**) If the distance (up down green arrow) between the skin surface and the contrast-enhanced lymph node (arrows) is measured beforehand, the SLN can be reached smoothly after the skin incision for SLN biopsy

In 2009, Omoto et al. [8] (24) reported CEUS with Sonazoid in a breast cancer patient. They then published the results of a preliminary clinical study on SLN detection using CEUS with subareolar Sonazoid injection in 20 breast cancer patients, in which the sensitivity of CEUS was 70%. In 2011, Omoto et al. [9] reported a further investigation of SLN identification with Sonazoid. A total of 181 breast cancer patients from four institutions were enrolled. After subareolar Sonazoid injection (Fig. 1), one or two contrast-enhanced LNs were detected in 166 of the 181 patients, with each taking 2 to 20 min to detect. Various flow patterns of lymphatic vessels (Fig. 2a–c) and contrast-enhanced SLNs (Fig. 3a, b) were shown.

In 2009, Sever et al. [10] reported that the overall sensitivity of CEUS-based SLN detection with SonoVue injected periareolarly was 89% (48 of 54 patients).

In 2017, Shimazu et al. [11] reported a feasibility study conducted at three hospitals. They showed that contrast-enhanced lymphatic vessels and SLNs were clearly visualized and easily identified with a detection rate of 98% (98/100) after Sonazoid injection periareolarly.

### Malignant melanoma

In 2009, Rue Nielsen et al. [12] reported subcutaneous injection of SonoVue on both sides of the scar from the excised tumor in ten malignant melanoma patients, but two contrast-enhanced inguinal LNs were visualized on CEUS in only one patient (10%).

### Oral and otopharyngeal cancer

In 2019, Wakisaka et al. [13] reported the successful detection of SLNs in 8 out of 10 cases after Sonazoid injection into the mucosa at the primary site in head and neck cancer patients (6 oral and 4 otopharyngeal cancers).

Clinical studies of SLN detection using CEUS in malignant cancer patients are presented in Table 2.

**Table 2:** Clinical study with CEUS for SLN identification in malignant cancer

Authors	Year	Type of cancer	Ultrasound contrast agent	Number of patients	Detection rate
Omoto K, et al. [7]	2006	Breast cancer	25% Albumin	23	100% (23/23)
Omoto K, et al. [8]	2009	Breast cancer	Sonazoid	20	70% (14/20)
Sever A, et al. [10]	2009	Breast cancer	SonoVue	54	89% (48/54)
Rue Nielsen K, et al. [12]	2009	Malignant melanoma	Sonazoid	10	10% (1/10)
Omoto K, et al. [9]	2011	Breast cancer	Sonazoid	181	92% (166/181)
Shimazu K, et al. [11]	2017	Breast cancer	Sonazoid	100	98% (98/100)
Wakisaka N, et al. [13]	2019	Oral and otopharyngeal cancer	Sonazoid	10	80% (8/10)

## Discussion

The SLN hypothesis states that tumor cells that are shed from a primary carcinoma migrate through a lymphatic channel to a single lymph node prior to the involvement of the remaining lymph nodes within that basin. Therefore, SLN identification and analysis for tumor involvement may predict the status of the remaining lymph nodes and provide important information preoperatively.

Much basic research in various fields has been reported in relation to SLN identification. In 1977, Cabanas et al. [14] reported inguinal lymphangiography, which is a radiological method, which confirmed the flow of contrast media into the SLN in penile cancer.

In 1992, Morton et al. [15] reported SLN detection and biopsy using the dye method in a clinical study of malignant melanoma. In breast cancer studies, Krag et al. [16] reported SLN biopsy using the radioisotope (RI) method with a gamma probe in 1993, and Giuliano et al. [17] reported the use of the dye method during surgery in 1994. Since then, these methods have been widely adopted all over the world.

Currently, SLN detection and biopsy using blue dye, RI with a gamma probe, or their combination have been performed in breast cancer with high identification rates [18]. However, anaphylactic reactions to the dye have rarely been reported [19, 20]. The radioisotope method usually requires more than several hours to detect SLNs after radioactive colloid is injected, leading to radiation protection problems.

In 2002, we developed and proposed a new identification method using a UCA to overcome these drawbacks. Initially, Omoto et al. [1] demonstrated its usefulness in animal experiments using 25% albumin solution as a UCA and obtained good results in a preliminary clinical study in breast cancer patients [7]. However, 25% albumin produced from human serum has been limited in use due to the risk of infection, and for this reason its use has not become widespread.

Starting with this 25% albumin solution [1, 7], many kinds of UCA such as hydroxyethylated starch [3], SonoVue [5, 21, 22], Sonazoid [2, 8, 9, 23], and Definity [4] have been utilized. With respect to SLN identification investigations using UCAs conducted in the last two decades, animal experiments are shown in Table 1 and clinical studies are shown in Table 2.

Among these UCAs, studies using Sonazoid have shown particularly good results in terms of SLN detection rates. The active ingredient of Sonazoid is perflubutane microbubbles, which have a long lifespan in the body because of chemical stability.

In addition to breast cancer, studies using SonoVue for malignant melanoma of the skin [12] and studies using Sonazoid for oropharyngeal cancer in the head and neck [13] have been reported. SLN detection with Sonazoid has shown a high identification rate in head and neck surgery, and it is also expected to be utilized in cancers other than breast cancer. Because CEUS is an imaging technique for detecting lymphatic channels and SLNs, it may be one of the most effective, versatile, and promising identification methods for SLNs.
