# Correction: Sentinel lymph node identification using contrast-enhanced ultrasound in breast cancer: review of the literature

**DOI:** 10.1007/s10396-024-01493-1

**Published:** 2024-08-23

**Authors:** Kiyoka Omoto, Kazushige Futsuhara, Tamami Watanabe

**Affiliations:** 1 Department of Laboratory Medicine, Jichi Medical University, Saitama Medical Center, 1-847 Amanuma-cho, Omiya-ku, Saitama, 330-8503 Japan; 2 Department of Surgery, Jichi Medical University, Saitama Medical Center, 1-847 Amanuma-cho, Omiya-ku, Saitama, 330-8503 Japan

**Correction: Journal of Medical Ultrasonics** 10.1007/s10396-023-01313-y

The article “Sentinel lymph node identification using contrast‑enhanced ultrasound in breast cancer: review of the literature”, written by Kiyoka Omoto, Kazushige Futsuhara and Tamami Watanabe, was originally published electronically on the publisher’s internet portal on 10 July 2023 without open access. With the society’s decision to opt for Open Choice the copyright of the article changed on 9 August 2024 to © The Author(s) 2023 and the article is forthwith distributed under a Creative Commons Attribution 4.0 International License, which permits use, sharing, adaptation, distribution and reproduction in any medium or format, as long as you give appropriate credit to the original author(s) and the source, provide a link to the Creative Commons licence, and indicate if changes were made. The images or other third party material in this article are included in the article’s Creative Commons licence, unless indicated otherwise in a credit line to the material. If material is not included in the article’s Creative Commons licence and your intended use is not permitted by statutory regulation or exceeds the permitted use, you will need to obtain permission directly from the copyright holder. To view a copy of this licence, visit http://creativecommons.org/licenses/by/4.0.

